# The Role of the Gut–Biliary–Liver Axis in Primary Hepatobiliary Liver Cancers: From Molecular Insights to Clinical Applications

**DOI:** 10.3390/jpm15040124

**Published:** 2025-03-24

**Authors:** Mario Romeo, Marcello Dallio, Fiammetta Di Nardo, Carmine Napolitano, Paolo Vaia, Giuseppina Martinelli, Pierluigi Federico, Simone Olivieri, Patrizia Iodice, Alessandro Federico

**Affiliations:** 1 Department of Precision Medicine, Hepatogastroenterology Division, University of Campania Luigi Vanvitelli, 80138 Naples, Italy; mario.romeo@unicampania.it (M.R.); fiammetta.dinardo@studenti.unicampania.it (F.D.N.); carmine.napolitano1@studenti.unicampania.it (C.N.); paolo.vaia@studenti.unicampania.it (P.V.); giuseppina.martinelli@studenti.unicampania.it (G.M.); simone.olivieri@policliniconapoli.it (S.O.); alessandro.federico@unicampania.it (A.F.); 2 Pharmaceutical Department, ASL NA3 Sud, Torre del Greco, 80059 Naples, Italy; federicopierluigi@libero.it; 3 Oncology Division, Monaldi Hospital, 80131 Naples, Italy; patrizia.iodice@ospedalideicolli.it

**Keywords:** cancer, hepatocellular carcinoma, cholangiocarcinoma, microbiota, metabolomics

## Abstract

**Background:** Hepatobiliary liver cancers (HBLCs) represent the sixth most common neoplasm in the world. Hepatocellular carcinoma (HCC) and cholangiocarcinoma (CC) constitute the main HBLC types, with alarming epidemiological projections. **Methods:** In recent decades, alterations in gut microbiota, with mutual implications on the gut–liver axis and gut–biliary axis permeability status, have been massively investigated and proposed as HBLC pathogenetic deus ex machina. **Results:** In the HCC setting, elevated intestinal levels of *Escherichia coli* and other Gram-negative bacteria have been demonstrated, resulting in a close association with increased lipopolysaccharide (LPS) serum levels and, consequently, chronic systemic inflammation. In contrast, the intestinal microbiota of HCC individuals feature reduced levels of *Lactobacillus* spp., *Bifidobacterium* spp., and *Enterococcus* spp. In the CC setting, evidence has revealed an increased expression of *Lactobacillus* spp., with enhanced levels of *Actynomices* spp. and *Alloscardovia* spp. Besides impaired strains/species representation, gut-derived metabolites, including bile acids (BAs), short-chain fatty acids (SCFAs), and oxidative-stress-derived products, configure a network severely impacting the progression of HBLC. **Conclusions:** In the era of Precision Medicine, the clarification of microbiota composition and functioning in HCC and CC settings can contribute to the identification of individual signatures, potentially providing novel diagnostic markers, therapeutic approaches, and prognostic/predictive tools.

## 1. Background

Hepatobiliary liver cancers (HBLCs) represent the sixth most common neoplasm in the world [1]. Hepatocellular carcinoma (HCC) (accounting for 80% of primary HBLCs) and cholangiocarcinoma (CC) (accounting for 10–20% of primary HBLCs) constitute the main HBLC types; considered together, they represent the most common incident cancers worldwide, with alarming epidemiological projections, particularly in Western countries [2,3]. Interestingly, several modifiable “industrialized-zone-associated” risk factors (including unhealthy lifestyle habits and related dysmetabolic features), largely found in Western areas, have been demonstrated to be shared by HCC and CC [2,3,4], making the identification of prevention strategies an urgent need in this setting. Both exogenous agents and endogenous genetic factors simultaneously contribute to cancer onset and progression; this complex scenario comprehensively describes the incompletely clarified multifactorial pathogenesis of HBLCs (both HCC and CC) [5,6,7,8,9].

In addition to genetic background, in recent decades, alterations in gut microbiota composition and functioning, with relative mutual implications for the “gut–liver axis” and “gut–biliary axis” permeability status, have been massively investigated as individual “signatures” and proposed as physiopathological deus ex machina impacting primary HBLC pathogenesis (in both cancer onset and progression), representing a novel potential therapeutic target and prognostic predictive tool [10].

Different systemic “therapeutic lines” have been developed and are currently available for advanced stages of HCC and CC [5,6]. However, despite recent considerable therapeutic progress [11], a significant portion of advanced HCC-affected and CC-affected patients continue to present with a poor prognosis, and non-negligible rates of serious adverse events to systemic treatment strategies have also been reported [3,12]. In this sense, the elaboration of models predicting the individual response [particularly for overall survival (OS) and progression-free survival (PFS)] to these regimens would have relevant repercussions for routine clinical practice, critically revolutionizing the tailored management of primary HBLC advanced-stage patients. In this scenario, from a translational point of view, the clarification of peculiar gut microbiota alterations in function and composition configuring a personal “signature” appears to be a significant contributor to guiding early diagnosis, developing personalized therapeutic strategies, and elaborating predictive tools, representing an ongoing open challenge.

Based on this background, the present review aims to explore the pathogenetic implications of the gut–biliary–liver axis in HBLCs, reporting the state of the art of the evidence on this topic and simultaneously showing potential novel approaches and applications in clinical practice.

## 2. Gut–Biliary–Liver Axis in the Pathogenesis of HBLCs

Alterations in the gut microbiota composition and functioning have been shown to critically influence the genesis and progression of several chronic liver disorders (CLDs), including hepatic malignancies [13]. Different pathogenic inflammatory immune-mediated mechanisms, sharing the impairment of the intestinal and biliary permeability as potential primum movens, have been progressively revealed, comprehensively sustaining and reinforcing the role of the “gut–biliary–liver axis” [13].

### 2.1. Impaired Intestinal and Biliary Permeability in the Pathogenesis of Hepatobiliary Liver Cancers

In physiological conditions, homeostasis of the gastrointestinal tract is guaranteed by maintaining an intact and effective barrier against intestinal bacteria, products of microbial origin [particularly lipopolysaccharides (LPSs)], and other potentially dangerous derivative products [14].

An adequate mucus layer representation overlying the proper epithelial cells’ conjunction, combined with a complex system of apical and basolateral structural/regulating proteins, constitute the crucial physiological elements determining the correct working of this barrier [14].

On the one hand, the major determinant of intestinal trans-epithelial transport is the claudin family of proteins, which allows, through their interactions, selective ion permeability by forming pores. On the other, scaffolding proteins create an essential complex to maintain epithelial integrity against potentially harmful luminal molecules by connecting the cytoskeleton to tight junctions (TJs) [14]. These molecules, including the zonula occludens (ZO) protein family (ZO-1, ZO-2, and ZO-3), TJ-associated MARVEL domain-containing proteins (TAMPs) (Occludin, MarvelD3, and Tricellulin), and junctional adhesion molecules (JAMs), link laterally adjacent cells near the apical surface, thus contributing to preserving the epithelial and mucus layers [15]. Besides the mucus stratus and the intestinal epithelium, the mucosal intrinsic layer (MIL) and the gut–vascular barrier (GVB), by regulating the improper translocation of bacteria and derivative products, represent further relevant components of this barrier [14].

In pathological scenarios, the disruption of the intestinal barrier (“leaky gut”) allows the intrusion of components into the portal (and subsequently systemic) circulation through chronically increased intestinal permeability, representing the potentially common denominator pathogenetic factor of various human disorders, with particular reference to CLDs and related malignancies [16].

In patients with CLDs, impaired LPS detoxification and endotoxemia clearance mechanisms have been largely revealed as consequences of massive bacteria (and derivative products) translocation into the portal circulation, significantly impacting liver disease progression and HBLC onset [13]. In this sense, the gut barrier disruption facilitates the translocation of LPS and other toll-like receptor (TLR) ligands to the liver via the portal vein [17]. Bacterial components, referred to as pathogen-associated molecular patterns (PAMPs), initiate inflammatory responses via activating specific TLRs in both the early and late stages of the disease, subsequently contributing to liver fibrosis and cirrhosis, which are essential substrates for HCC onset [17].

In parallel to the instauration of such “pro-cancerogenic” scenarios (i.e., liver fibrosis and cirrhosis), where liver injury-induced hepatic cell regeneration favors per se malignancy onset, a direct role of TLR (TLR-4 and TLR-2, overall) activation in HCC pathogenesis has been hypothesized and evaluated in different studies [18,19,20,21].

Concerning this, very recently, Wang X et al. revealed that fecal microbiota transplant (FMT)-containing stool samples from HCC patients spontaneously promoted liver inflammation, fibrosis, and dysplasia in wild-type mice, worsening disease progression in a mouse model of HCC [22].

The authors highlighted HCC-FMT results in gut barrier injury and translocation of live bacteria to the hepatic microenvironment, evidencing particularly an enrichment of *Klebsiella pneumoniae* (*K. pneumoniae*) [22]. At the hepatic level, the gut-derived pathogen *K. pneumoniae* surface protein PBP1B interacts with and activates TLR4 on HCC cells, leading to increased cell proliferation and activation of oncogenic signaling, whereas TLR4 inhibition represses *K. pneumonia*-induced HCC progression [22].

These results indicated that PAMPs-TLR pathways promote carcinogenesis, highlighting the crucial role of other gut-derived TLR ligands beyond the “classic” LPS. In support of this evidence, other research has suggested that TLR activation in non-bone-marrow-derived resident liver cells promotes cancer progression by enhancing inflammation, impacting immunosurveillance, as well as fostering proliferative and antiapoptotic signals [18,19,20,21].

Concerning this, Miura K et al., using hepatocyte-specific Pten-deficient [Pten(Δ) (hep)] mice models exhibiting steatohepatitis followed by HBLC onset (including HCC)], generated Pten(Δ) (hep)/Tlr4(-−/−) mutant mice and investigated the role of macrophages using reconstitution of bone marrow (BM)-derived cells, chemical depletion of macrophages, and isolated macrophages [18]. In this study, the authors reported several relevant findings: (a) Tlr4 deficiency in the Pten(Δ) (hep) mice suppressed tumor growth as well as hepatic inflammation; (b) gut sterilization by an antibiotic mixture reduced the portal LPS levels as well as tumor growth in the Pten(Δ) (hep) mice; c) HCC growth was also decreased by reconstitution of BM-derived cells to Tlr4(−/−) BM cells; d) chemical depletion of macrophages significantly reduced tumor size and numbers; and e) hepatic macrophages isolated from the Pten(Δ) (hep) mice presented an enhanced production of proinflammatory cytokines [interleukin (IL)-6 and Tumor Necrosis Factor-alpha (TNF-alpha)] in response to LPS promoting the HCC proliferation [18]. Altogether, these findings demonstrated that the potential action of gut microbiota-derived PAMPs (including LPS) as activators of hepatic TLR4 contributes to the development of steatohepatitis-related HCC in the mouse via macrophage-mediated inflammation [18].

In line with this, Cowden et al. tested two inhibitors of the histamine H4 receptor interacting with TLR4, revealing a reduced TNF-α production and LPS-induced inflammation in mouse livers [20]. In recent in vivo/in vitro research, Esparza-Baquer et al. investigated the impact of the triggering receptor expressed on myeloid cells 2 (TREM-2) (which is predominantly expressed in hepatic non-parenchymal cells and inhibits TLR signaling) on liver regeneration and hepatocarcinogenesis [23]. The authors, by assessing TREM-2 expression in liver tissues of two independent cohorts of patients with HCC, comparing it with control liver samples, and performing in vitro studies with hepatic stellate cells (HSCs) and HCC spheroids, highlighted an upregulation of TREM-2 expression in human HCC tissue, also evidencing conditioned media from HSCs overexpressing TREM-2-inhibited HCC spheroid growth through attenuated Wnt/β-catenin ligand secretion [23].

Other findings have revealed a positive correlation between the activation of hepatic TLR-2 and proliferation, vascularization, and extra-hepatic diffusion in HCC [19,21]. Regarding this, Zhe et al., in a study performed on Huh-7 HCC cells, initially demonstrated in vitro the role of extracellular HSP70-peptide complexes in promoting the proliferation of HCC cells via the TLR2/Mitogen-activated Protein Kinase (MAPK) pathway [21].

More recently, in research performing immunohistochemistry analyses on liver tissue HCC microarrays, a significant correlation of TLR-2 expression with proliferative index Ki67 (r: 0.24), Caspase-3 expression (r: 0.27), and vascularization (r: 0.56) was reported [19]. In addition, treatment with a TLR-2 agonist of Huh-7 HCC cells induced the expression of cellular proliferation (CD34) and vascularization markers (VEGF) [19].

Altogether, the above-presented findings configure a pathogenetic context where the liver appears as a two-faced Janus, simultaneously representing the first target organ receiving physiological gut-derived products as well as, in the case of impaired intestinal permeability, potentially dangerous microbe-derived products contributing to hepatic disorders and cancerogenesis [16].

In this scenario, besides intestinal integrity, growing evidence suggests the role of biliary permeability in human diseases, thus proposing the liver as a “second firewall”. In this sense, to maintain homeostasis in the hepatic interstitial tissue, it is essential to preserve the TJs in both the hepatocytes of the canaliculi and the cholangiocytes of the bile duct [24]. As a consequence, therefore, biliary permeability may potentially play an equally fundamental role in the pathogenesis of liver diseases and HBLCs.

However, unlike the well-documented structure and regulation of intestinal TJs [16], the knowledge concerning the biliary tract is limited. In murine models, immunofluorescence has been used to evaluate the linear distribution of Occludin and ZO-1 throughout the canalicular pole, and the biliary barrier function was closely associated with the co-localization of Occludin and ZO-1 in the intercellular space [24].

Regarding the regulatory mechanisms of biliary TJ function, it has emerged that Occludin is hyperphosphorylated on Serine (Ser) and Threonine (Thr) residues in intact epithelial TJs, while phosphorylation on Tyr residues occurring in situations of damage results in the loss of its interaction with ZO-1, ZO-2, and ZO-3 and can worsen biliary permeability [24]. Relevantly, oxidative stress and inflammatory mediators such as bacterial LPS can impact this, inducing dephosphorylation of threonine residues on TJs and modulating a migration of the proteins that constitute these junctions from the intercellular to the intracellular compartments [25]. On the other hand, protective factors preserving the barrier action of the bile duct epithelium have been recognized, such as the epidermal growth factor (EGF), by reducing the hydrogen peroxide-induced disruption of TJs [26].

The question of whether the alteration of these mechanisms is primarily involved in the disease pathogenesis or if this occurs secondarily in the pathological course remains unanswered. Nevertheless, the compromise of these structures is a recurring theme in several hepatobiliary diseases, including HBLC, and the carcinogenesis process affects TJs by regulating the expression of their components in many ways [27].

In addition, relevantly, each cancer type shows a sort of “signature expression” in the TJ proteins that could also potentially help in the diagnosis process [28]. Regarding this, Patonai et al. revealed overexpression of claudin 3, 4, and 7 in CC and an undetectable one in fibrolamellar HCC, while Tricellulin was downregulated in all cancer types compared with the normal liver [28]. Moreover, this disruption also has a possible role in the metastatic process, as shown by the significant reduction of cell migration and invasion in CC cell lines after claudin 4 suppression [29].

The gut microbiota appears to be actively involved in preserving intestinal and biliary permeability, and consequently, alterations in the relative composition and functioning have been reported to determine negative repercussions on the integrity of this bulwark [14,30]. In this sense, one of the primary physiological goals of gut bacteria–epithelial cell interactions is to finely regulate permeability through the modulation of TJs [14,30].

In this setting, in addition to the importance of an effective barrier system capable of preventing leaky gut-related scenarios, the preservation of adequate intestinal microbiota composition plays an equally fundamental role [31]. Indeed, as a vicious circle, the impairment of intestinal permeability, besides favoring bacterial and derivative products’ translocation, also promotes bacterial overgrowth with crucial changes in the composition of the microbiota, significantly contributing to the shift from the physiological state of “eubiosis” to the pathological state of “dysbiosis” [31].

Relevantly, similar to the above-reported reciprocal influence of altered gut microbiota composition and impaired intestinal permeability [31], a mutual pathogenetic relationship has also been proposed for dysbiosis and liver cancer, suggesting the capability of dysbiosis to impact HBLC progression as well as the repercussions determined by the hepatic tumoral microenvironment on gut microbiota species’ representation and functioning [32,33]. These implications in HBLC (both HCC and CC) pathogenesis are discussed in detail in the following subparagraphs of this review.

### 2.2. Principal Alterations in Gut Microbiota Composition in Hepatobiliary Liver Cancers

#### 2.2.1. Altered Gut Microbiota Composition and Hepatocellular Carcinoma

An imbalance in gut microbiota composition has been largely reported in patients affected by HCC [34,35,36]. In this setting, comprehensively, elevated levels of *Escherichia coli* and other Gram-negative bacteria have been demonstrated in the intestinal flora, resulting in a close association with increased serum LPS levels/endotoxemia and, consequently, chronic systemic inflammation [37]. On the other hand, the intestinal microbiota of HCC individuals features reduced levels of *Bifidobacterium* spp. and *Enterococcus* spp. [34,35,36].

This relevant translational evidence has been initially investigated in murine models [35,36]. Firstly, Zhang et al. revealed the administration of probiotic-mitigated dysbiosis and decreased liver tumor growth via impacting endotoxemia; the presentation of PAMPs; and, strikingly, the activation of endogenous damage-associated molecular patterns (DAMPs) [like high-mobility group box 1 (HMGB1)], ultimately contributing to reduced chronic inflammation in the hepatic microenvironment [36].

Subsequently, on the same line, Schneider et al. revealed that gut dysbiosis influences antitumor immune surveillance and drives liver disease progression toward cancer via interfering with inflammasome-mediated pathways [35]. In Nucleotide-binding oligomerization domain Leucine-rich Repeat Pyrin domain containing (Nlrp6) (-/-) mice, the dysbiotic microbiota was shown to induce a TLR4-dependent expansion of hepatic monocytic myeloid-derived suppressor cells (mMDSC) simultaneously with the suppression of T-cell abundance [35].

Relevantly, highlighting the large plasticity of the tumor microenvironment, in this work, the transmissibility of this phenotype via FMT and the reversibility by antibiotic regimens were also reported, particularly remarking on the functional role of *Akkermansia muciniphila* [35]. Consistently, emerging research has recently focused on this bacterium given that the reduction of *Akkermansia muciniphila* appears to be associated with the abundance of mMDSCs, with consequent suppression of the anti-tumoral immune response; conversely, its supplementation ameliorates liver injury, inflammation, and fibrosis and induces a significant shift in microbiota composition in murine models [35]. In particular, this reintroduction leads to an abundance of *Lachnospiraceae* and *Blautia*, which have demonstrated anti-inflammatory properties due to the production of short-chain fatty acids (SCFAs) [38].

Similarly, in more recent human research conducted on patients with HCC, Behary et al. specifically focused on characterizing gut microbiota in individuals with cirrhosis related to non-alcoholic fatty liver disease (NAFLD) (recently renamed Metabolic Dysfunction-Associated Steatotic Liver Disease—MASLD) [39], with or without HCC, evidencing dysbiosis as a recurrent feature in subjects with MASLD-cirrhosis, with compositional and functional shifts occurring with HCC development, particular regarding the metabolomic analyses-assessed production of SCFAs [40]. In this setting, the pathogenetic implications of SCFAs in hepatic cancerogenesis are detailly described in the dedicated section (“2.3.2 Short-chain fatty acids (SCFAs) in hepatocarcinogenesis”) of this review.

MASLD represents a predominant hepatopathy in Western countries, embracing a spectrum ranging from simple steatosis to steatohepatitis and advanced fibrosis/liver cirrhosis [39]. Relevantly, in recent decades, growing evidence has alarmingly supported the potential onset of HCC even in the initial stage (simple steatosis or steatohepatitis) of MASLD, dramatically remarking on the non-exclusivity of hepatic cancerogenesis processes in the advanced fibrosis/liver cirrhosis context, which significantly complicates the adoption of effective screening strategies for these patients in routine clinical practice [41]. Therefore, research efforts have focused on identifying pathogenetic factors potentially contributing to this scenario, including alterations in the gut microbiota composition. On this topic, previous studies highlighted intestinal dysbiosis even in MASLD patients with simple steatosis, particularly reporting an increased relative abundance of *Proteus* and *Enterobacter* bacteria, simultaneously with decreased levels of *Ruminococcus* and *Lactobacillus* [42,43]. Interestingly, as simple steatosis progresses to steatohepatitis and advanced liver fibrosis, an increased abundance of Gram-negative species is observed, with a particular reference to *Proteus* bacteria [44,45].

However, although these studies suggest a potential pathogenetic correlation between the gut microbiome composition and primary HCC in the MASLD-related setting in different liver disease progression stages, the causal relationship remains unclear because of confounding factors, without also overlooking reverse causality [38]. Concerning this last feature, indeed, the liver structure also appears to be a factor potentially influencing gut microbiome diversity in HCC individuals, given that an imbalanced composition of microbial flora has been reported to be more prevalent in patients with HCC and cirrhosis than in those with HCC without cirrhosis, functionally leading to decreased SCFA-producing and increased LPS-producing genera [46,47].

In particular, the *Streptococcaceae* family and the *Lactococcus* genus appear significantly higher in the gut microbiota of cirrhotic HCCs than non-cirrhotic HCCs [46].

Furthermore, as a chain reaction effect, hepatic cirrhosis-affected patients show increased bacterial abundance in hepatic tissue, which induces pronounced transcriptional changes, including activation of fibro-inflammatory pathways as well as circuits mediating cancer immunosuppression [35,46]. Consistently with this, both *Streptococcaceae* and *Lactococcus* taxa are also increased in HCC tissues in comparison to normal liver tissues, suggesting a role in liver fibrosis and carcinogenesis [46]. Conversely, the phylum *Verrucomicrobiota*, class *Chlamydiae*, orders *Xanthomonadales* and *Caulobacterales*, family *Caulobacteraceae*, and genus *Bradyrhizobium* have been reported to be diminished in cirrhotic HCC tissues according to fresh tissue cultures [46].

Looking beyond the MASLD setting, Komlyama et al. stratified these findings according to the CLD etiology in patients with HCC; amplicon sequence variants (ASVs) were significantly greater in the tumor region than in the non-tumor region in both viral and non-viral (i.e., MASLD) settings [48]. Moreover, *Ruminococcus gnavus* from the *Lachnoclostridium* genus, a prevalent member of the “normal” human gut microbiota, was found in viral HCC patients but not in non-viral HCC, suggesting a taxonomic signature and a possible role in the pathogenesis of this cancer [48].

More recently, a Mendelian randomization (MR) study was conducted using summary statistics from genome-wide association studies (GWAS) of the gut microbiome and liver cancer and sequencing data from a case-control study including HCC patients and healthy controls [49]. Relevantly, in GWAS, protective causal associations with HCC for the *Ruminococcaceae* family (OR: 0.46) and *Porphyromonadaceae* genus (OR:0.59) were revealed, confirming these results in the case-control study, which showed a higher relative abundance of these bacteria in controls than HCC patients [49].

In addition to this, modern evidence suggesting the possible migration of oral microorganisms to the gut (directly, via the digestive tract, or by entering the circulatory system and subsequently colonizing the bowel) further complicates the above-presented pathogenetic scenario [50]. This translocation is physiological and fundamentally sustains the “oral–gut” axis [50].

As a domino effect, alterations in oral microbiota can affect the gut microbiome and thus gut-dysbiosis-associated human diseases, including HCC [50,51]. Concerning this, Lu et al. first investigated the tongue coat microbiome of patients with HCC and cirrhosis using 16S ribosomal RNA (rRNA) gene sequencing in patients with HCC and healthy subjects [34]. The authors reported substantial microbiome diversity in the tongue coat of HCC patients, and according to their analysis, *Oribacterium* and *Fusobacterium* could distinguish these patients from healthy individuals, transitionally providing a novel and non-invasive potential diagnostic tool for hepatic cancer [34]. However, despite growing findings for potentially useful microbiome-related salivary markers in other disorders and the initial promising perspectives, no research has subsequently investigated this topic in the HCC setting specifically, and no practical clinical applications have been derived until very recently Artificial Intelligence (AI)-based models have provided new information in this field (as detailly reported in the clinical applications-dedicated section of the present review) [34].

In conclusion, the clarification of the alterations of microbiome composition interesting the whole human intestinal tract (including the oral district), simultaneously characterizing the relative functional pathogenetic implications, still represents an open challenge in elucidating the pathogenetic mechanisms of HCC.

#### 2.2.2. Altered Gut Microbiota Composition and Cholangiocarcinoma

Considering the current literature concerning HBLC pathogenesis, although precise mechanisms sustaining the implications of gut microbiota in the pathogenesis of this cancer have not been completely elucidated, non-negligible evidence suggests intestinal flora may also play a role in the genesis and progression of both intrahepatic (ICC) and extrahepatic (ECC) CC [52,53,54].

In support of this, initially, gut dysbiosis has been highlighted in precancerous diseases of CC, particularly the primary sclerosing cholangitis (PSC) setting and liver fluke infections [52]. In animal evidence first, and in human research subsequently, the potential mechanisms of biliary carcinogenesis related to gut dysbiosis have been proposed [49,53,55].

In the PSC setting, Zhang et al. reported a crucial role of intestinal flora composition in regulating anticancer immunity in CC by controlling the hepatic accumulation of polymorphonuclear myeloid-derived suppressor cells (PMN-MDSCs) through the TLR4-dependent mechanism and the chemokine (C-X-C motif) ligand 1 (CXCL1)/C-X-C chemokine receptor 2 (CXCR2) axis, highlighting a relevant implication of dysbiosis in promoting carcinogenesis in mouse models [55]. In particular, the activity of PMN-MDSCs is paramount in fueling cancer progression via inducing immune escape mechanisms, as these immune cells suppress cytotoxic T lymphocytes, enhancing neo-angiogenesis, vascular invasion, and metastasis [56,57]. On the other hand, in line with the concepts reported in the previous section concerning TLRs, a positive association of TLR4 activation with CC worsening has been highlighted, whereas lower TLR4 levels were associated with reduced cancer growth [58]. Once again, these findings remark on the cruciality of preserving an adequate intestinal barrier, as altered microbiota and/or gut–barrier permeability disruption promotes LPS translocation in the hepatobiliary tract, leading to TLR4 activation and ultimately sustaining chronic inflammation, cancerogenesis, and immune escape via reducing mononuclear antitumor functionality [10,27,54]. In support of this, in the same study, Zhang et al. also reported a higher concentration of PMN-MDSCs in the liver samples of PSC-affected patients with active Ulcerative Colitis (UC) compared both to individuals presenting PSC with inactive UC and PSC-affected subjects without UC, confirming the disruption of the intestinal barrier featuring active UC affected patients as a pathogenetic primum movens promoting the translocation of bacteria and LPS to the liver [55].

Focusing on liver flukes, *Opisthorchis Viverrini* infection has been widely shown to be a CC risk factor inducing an alteration of the gut microbiota composition associated with an increase in *Helicobacter* spp. in stool samples from these individuals [53,59,60].

In line with this, a microbiota analysis of tumor samples from CC patients showed an increase in three *Helicobacter* spp., including *H. pylori*, *H. bilis*, and *H. hepaticus*, as well as increased Ki67 levels, indicating exalted cell mitosis in these samples [61,62]. Consistently, in vitro research previously revealed an elevated expression of antiapoptotic factor B-cell lymphoma 2 (bcl-2), simultaneously with enhanced activation of MAPK and nuclear factor kappa-light-chain-enhancer of activated B cell (NF-kB) pathways, in CC cells cultured with Helicobacter spp. CagA+, thus sustaining bile duct cancer cells’ uncontrolled proliferation and survival [63].

Comprehensively, these findings reveal, besides the above-mentioned phlogosis-mediated mechanisms, the crucial role of alterations in the composition of gut microbiota in promoting cancerogenesis via interfering with biliary cell proliferation, survival pathways, and functioning [53].

Focusing on MASLD, hepatic steatosis simultaneously with insulin resistance (IR)-related cardiometabolic risk factors (CMRFs) configuring metabolic dysfunction, with a particular reference to associated Diabetes Mellitus type 2 (T2DM), constitutes a well-recognized high-risk scenario for ICC, justifying the recent epidemiological data revealing the rising incidence of HCC in parallel with CC in this setting [54].

Moreover, consolidated evidence supports the mutual relationship between T2DM (and relative medications) with dysbiosis, highlighting the relative implication of the altered gut microbiota composition in influencing the progression of liver disease through both (dys-)metabolic and inflammatory mechanisms [64].

The reduction of SCFA-producing species and the increased representation of LPS-releasing species, simultaneously with inflammation properly driven by IR, synergistically contribute to CC onset and progression at the hepatic level, especially in simple steatosis/steatohepatitis MASLD patients presenting with bile duct involvement [65].

Therefore, in line with previous-reported evidence for HCC, as simple steatosis progresses to steatohepatitis and advanced liver fibrosis, gut microbiota also dynamically changes for CC [44,45,64].

Considering these shared contact points in HBLC pathogenesis, identifying gut microbiota composition-related features able to discriminate HCC from CC represents a crucial research challenge with relevant practical repercussions.

Regarding this, a recent Mendelian randomization (MR) study conducted using summary statistics from genome-wide association studies (GWAS) of the gut microbiome and liver cancer, based on sequencing data from a case-control study including HCC patients, ICC individuals, and healthy subjects, revealed a protective causal association exclusively with ICC for the *Porphyromonadaceae* family (OR: 0.36) and *Bacteroidetes* genus (OR:0.59), confirming these results in controls, who had a higher relative abundance of these bacteria than CC patients [49].

Moreover, unlike HCC patients showing reduced levels of these species [34,35,36], compared with healthy individuals, an increased expression of *Lactobacillus* spp. has been reported in patients with CC [53]. This difference in composition is associated with functional pathogenetic repercussions, thus contributing to determining a specific signature potentially differentiating HBLCs (Table 1).

Besides *Lactobacillus* spp., enhanced levels of *Alloscardovia* spp. have also been highlighted in CC patients, critically impacting secondary BA metabolism (see next section) [53].

Altogether, the above-presented evidence suggests the concrete need to further elucidate the alterations of microbiome composition in the whole human intestinal tract, simultaneously featuring relatively functional implications, and not merely limiting to reporting the different bacteria distribution representation in healthy subjects compared with HBLC-affected individuals [70]. In line with this, besides these above-illustrated classical mechanisms, novel findings support the role of gut microbiome-derived metabolites (“gut microbial metabolites”) in significantly influencing HBLC pathogenesis via conditioning systemic/local inflammation, immune pathways, and cell proliferation mechanisms [10,40]. The relative state of the art on this emerging topic is presented in the next dedicated paragraph of this review.

### 2.3. Gut Microbial Metabolites in the Pathogenesis of Primary Hepatobiliary Liver Cancers

Gut dysbiosis-sustained intestinal permeability impairment represents the first piece of a pathogenetic domino opening to further extra-hepatic physiopathological events, ultimately contributing to the occurrence of HBLCs via the genesis of a pro-phlogistic, pro-oxidative, and immunosuppressive microenvironment in the liver [10,71]. In this “leaky gut” context, a significant shift in the intestinal production of specific gut-derived metabolites is promoted and has been largely reported with specific reference to bile acids (BAs) and short-chain fatty acids (SCFAs) [10,72]. Relevantly, as a “butterfly effect”, considering the underlying impaired permeability, these changes may have crucial repercussions at the hepatic level, contributing to aberrations in cell survival and abnormalities in cell proliferation mechanisms by simultaneously sustaining inflammation, alterations in the immune response, and oxidative stress [10].

#### 2.3.1. Bile Acids (BAs) in Hepatobiliary Carcinogenesis

Among metabolites potentially involved in carcinogenesis, BAs are currently receiving increased attention due to their known tumor-promoting properties, widely emerging as potential modulators of fundamental cancer-related processes impacting cellular phenotypes, including senescence, proliferation, as well as the epithelial-mesenchymal transition [72,73].

Physiologically, BAs are mainly synthesized by the liver (“primary BAs”) and, from a purely immunological point of view, generically exert direct or indirect antimicrobial effects on the whole gut–biliary–liver axis, acting as an innate defense mechanism against bacterial infections [74,75].

Emerging evidence suggests alterations in the primary BA pool as a crucial pathogenetic moment contributing to hepatic carcinogenesis through the disruption of various signaling pathways [including Janus Kinase-Signal Transducer and Activator of Transcription (JAK-STAT3), cyclooxygenase-2 (COX-2), and NF-kB], amplifying the polarization of M2-like tumor-associated macrophages (TAM-M2), and enhancing the local production of inflammatory mediators [IL-6, IL-1beta, and TNF-alpha] via activating inflammasome [76,77]. As a vicious circle, in turn, these cytokines interfere with apoptotic processes by engaging the JAK-STAT3 and Phosphatidylinositol 3-Kinase (PI3K) pathways, promoting cell survival and potentially leading to cell immortalization [76], as well as creating a pro-tumor microenvironment where the reduced function of CD8+ T cells and recruitment of natural killer T (NKT) cells favor tumor progression via fostering immune escape [78].

In this scenario, the gut microbiota plays a pivotal role in influencing both physiological and pathological processes [10,13]. Interestingly, the relationship between the gut microbiota and BAs indeed appears bidirectional: while the microbial flora influences primary BAs’ metabolism and synthesis, the BA pool and composition influence the diversity and the homeostasis of the gut microbiota [79]. Furthermore, relevantly, the intestinal flora is also directly responsible for certain BA species production known as “secondary BAs” [73,80].

In the large intestine, a fraction of primary BAs is converted to secondary BAs by gut bacteria [73,80]: in the colon, the gut microbiota helps remove water from hepatic-derived BAs and is also involved in their deconjugation, dihydroxylation, and dehydrogenation [80]. More specifically, *Bacteroides*, *Clostridium*, *Lactobacillus*, *Bifidobacterium*, *Listeria*, and *Escherichia* species participate in deconjugation through bile salt hydrolase activity.

The unconjugated primary BAs are then further dehydrogenated and 7α-dehydroxylated mainly by *Clostridium* species, resulting in the formation of secondary BAs [73,80]. In the end, a large portion of the secondary BAs undergo hepatic reuptake, whereas a small fraction of intestinal BAs remain in the circulation and exert systemic effects [80].

Consequently, alteration of certain secondary BA levels depending on the impaired representation of specific microbial species appears conceivable, creating a context where the impaired permeability can potentially influence the hepatic afflux of these compounds, and simultaneously, the shift in these gut-derived metabolites may impact cancer onset and progression via different mechanisms [10,13].

In cases of dysbiosis, a considerable increase in the levels of deoxycholic acid (DCA), a secondary BA, has been reported, contributing to liver injury; abnormal hepatocyte proliferation; and, ultimately, the promotion of HBLCs through the suppression of farnesoid X receptor (FXR) [10]. At the hepatic level, FXR represents the most important BA metabolism-regulating nuclear receptor, mainly controlling the activation, among several others, of the Wnt/β-catenin signaling pathway, appearing to thus be critically involved in the process of hepatocarcinogenesis with particular reference to malignant cell proliferation and migration [81]. Consistently, the inhibition of FXR functions has been associated with DNA damage and impaired cell proliferation, and the loss of FXR has been markedly correlated with aggressive tumor phenotypes and poor prognosis in patients with HCC and CC [81,82], while relative activation by FXR agonist (obeticholic acid) represents a promising anti-cancerogenic frontier to explore in the field of hepatic tumors [81,83]. In CC clinical tissues, the expression of FXR has been negatively correlated with IL-6 levels, and the activation of FXR by obeticholic acid inhibits tumor growth and metastasis via IL-6 suppression [82]. Interestingly, in line with this, another in vivo study revealed the role of DCA in the upregulation of the NF-kB pathway, thus promoting the overexpression of IL-6 in the HCC setting [84].

Focusing on the same IL-6 pathway, in the presence of the enhanced intestinal relative abundance of *Ruminococcaceae*, Jia et al. reported higher levels of further secondary BAs, including glycochenodeoxycholic acid (GCDA), among others [taurocholic acid (TA), glycodeoxycholic acid (GDA), tauroursodeoxycholic acid (TUDCA), and taurodeoxycholic acid (TDA)], preceding a decreased concentration of chenodeoxycholic acid (CDA) and increased serum levels of IL-6 in CC patients presenting vascular invasion [54]. In line with this, in CC, increased GCDA levels have been demonstrated to fuel chronic inflammation via induction of endoplasmic reticulum stress, promoting increased release of reactive oxygen species (ROS), DNA damage, and ultimately aberrating cell replication [85,86]; furthermore, reduced CDA levels have been shown to stimulate cell proliferation via interfering with EGFR/Early growth response factor 1 (EGR1)/MAPK and protein kinase C (PKC)/MAPK/NF-kB signaling [87].

Consistently, other evidence supports the role of CDA, simultaneously with different alterations in the levels of other hydrophobic secondary BAs [e.g., cholic acid (CA), glycocholic acid (GCA), and lithocholic acid (LA)], in contributing to chronic inflammation and interfering with apoptosis via influencing Fas death receptor signaling, cytochrome c release, and caspase 9 activation pathways [87,88].

Besides the enhanced relative abundance of *Ruminococcaceae*, in CC patients, increased levels of *Lactobacillus* spp. and *Alloscardovia* spp. have also been reported, critically impacting secondary BA metabolism [53]. Regarding this, in the same above-mentioned research, Jia et al. also highlighted a positive correlation between *Lactobacillus* and *Alloscardovia* genera and TUDCA plasma–stool ratios, suggesting their possible role in discriminating CC presenting with vascular invasion [54]. Moreover, plasma TUDCA levels were negatively correlated with *Pseudoramibacter* abundance and CC survival time [54].

Altogether, this evidence highlights the cruciality of the gut dysbiosis-induced shift in BA production with dramatic repercussions for HBLC onset and progression via impacting, through influencing tumor-associated proinflammatory cytokines production and local oxidative status, the functioning of several major receptor-regulated cell proliferation, survival, and migration pathways [10,89].

**Figure 1:**
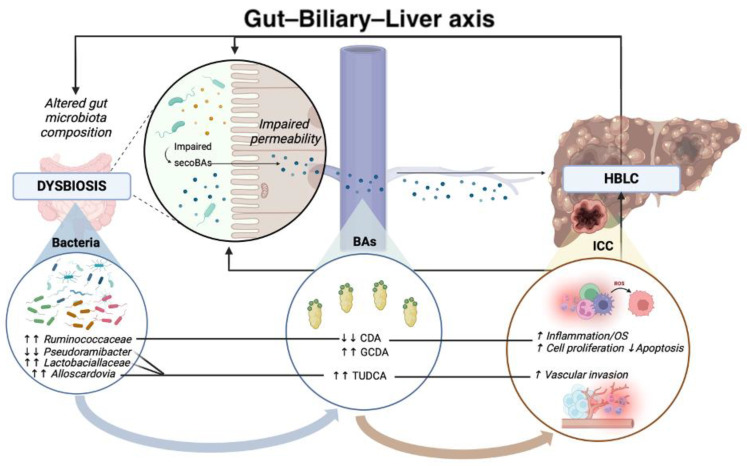
Principal gut dysbiosis-related alterations in the secondary biliary acids pool contributing to HBLC progression. Abbreviations: BAs: bile acids; HBLC: hepatobiliary liver cancer; ICC: intrahepatic cholangiocarcinoma; CDA: chenodeoxycholic acid; GCDA: glycochenodeoxycholic acid; TUDCA: tauroursodeoxycholic acid. (↓↓): significantly reduced levels; (↑↑): significantly increased level. (↑): increased level; (↓): reduced level.

Figure 1 summarizes the most relevant implications of intestinal dysbiosis-determined alternated secondary BA levels in the pathogenesis of HBLC (Figure 1).

#### 2.3.2. Short-Chain Fatty Acids (SCFAs) in Hepatobiliary Carcinogenesis

Physiologically, SCFAs (mainly represented by acetate, butyrate, and propionate) are generated by the gut microbiota through the fermentation of non-digestible carbohydrates [90]. These molecules play a pivotal role in gut homeostasis by preserving the stability of the intestinal epithelial barrier, simultaneously contributing to preserving the adequate diversity of the gut microbiota [70,90]. Consequently, in pathological conditions altering SCFAs’ production, these species have been reported to mutually impact dysbiosis as well as influence gut barrier permeability, intestinal epithelial cell metabolism, immune response, carcinogenesis, and tumor progression in various oncological settings, including HBLC, with a particular reference to acetate and butyrate [70,90].

Acetate has been shown to inhibit cancer progression by suppressing group 3 innate lymphoid cell (ILC3) infiltration in tumoral tissue, whose negative correlation with a negative prognosis in HCC has been demonstrated [67]. Recently, Hu et al. reported a severe reduction of *Lactobacillus reuteri* in the gut microbiota of mice with HCC, accompanied by decreased SCFA levels, especially acetate [67]. The same study suggested acetate reduced the production of IL-17A in hepatic ILC3s by disrupting histone deacetylase (HDAC) activity. The authors also revealed that the combination of acetate with the programmed death 1/programmed death ligand 1 blockade significantly enhanced antitumor immunity [67]. These findings are consistent with other research sustaining the cruciality of acetate in anti-cancerogenesis via antagonizing immunosuppression mediated by DNA epigenetic modifications, particularly disrupting the HDAC activity, a well-known crucial regulator of gene transcription, whose dysregulation in cancers has been widely reported [91,92].

In contrast, butyrate has demonstrated conflicting roles in HBLC, emerging initially as a factor promoting tumor progression in some studies and as an effective anti-cancer mediator and enhancer of the efficacy of immunotherapy in several others [93,94,95,96].

Relevantly, stool analyses of patients with HCC showed a reduced presence of SCFA-producing bacteria like *Lachnospiraceae*, *Ruminococcaceae*, and *Butyricicoccaceae*, representing the main producers of butyrate [97]. In vitro studies also demonstrated that CC cells treated with butyrate showed a positive effect on cilia formation and acetylated tubulin levels, reducing cell mitosis [10].

On the other hand, dysregulation in the butyrate levels (and gut microbiota-derived SCFAs in general) in terms of excessive production may significantly contribute to an immunosuppressive hepatic microenvironment through the exasperated activity of T-regulatory cells and IL-10 secretion, and thus to HBLC by ultimately promoting immune escape [33]. In line with this, Behary et al. recently characterized gut microbiota in patients with NAFLD-related cirrhosis with or without HCC and, through metagenomic and metabolomic analyses, investigated the relative effect on the peripheral immune response [40]. In this study, an ex vivo analysis revealed that bacterial extracts from the NAFLD-HCC microbiota, but not from the control groups, elicited a T cell immunosuppressive phenotype characterized by expansion of T-regulatory cells and attenuation of CD8+ T cells. Relevantly, the microbiota gene function in NAFLD-HCC supported SCFAs’ production, and this was confirmed by metabolomic assessments [33].

Furthermore, other research has linked aberrant SCFA production to the onset of hepatic cancer. In mice with high BAs, feeding them inulin to boost SCFA production resulted in increased liver inflammation, neutrophil infiltration, and a higher risk of HBLC [98].

Altogether, these emerging findings suggest the relevance of abandoning the classic absolutist conception of SCFAs as desirable gut-derived metabolites in every scenario and leaving space for a modern view considering their dysregulation, even in terms of excessive levels, as a possible contributor to HBLC onset and progression [98]. At the same time, the above-presented evidence remarks on the cruciality of comprehensively investigating gut-derived metabolites, without focusing on single bacteria or molecule species, given that the complex interplay between HBLC and dysbiosis is manifested through the simultaneous and reciprocally influenced alteration in the expression levels of several metabolites (SCFAs, BAs, etc.) composing an imbricated pathogenetic network [10,98].

**Figure 2:**
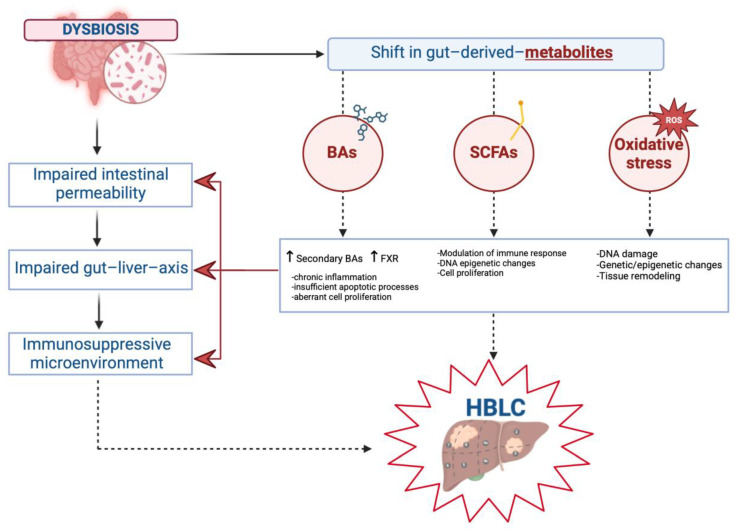
Gut–liver axis derangement and changes in metabolome sustaining HBLC. Abbreviations: BAs: bile acids; SCFAs: short-chain fatty acids; FXR: farnesoid X receptor; HBLC: hepatobiliary liver cancer. (↑): increased level.

Figure 2 summarizes gut–liver axis derangement and relative changes in the metabolome involved in HBLC onset and progression (Figure 2).

The above illustration highlights the role of gut dysbiosis in determining an impairment of intestinal permeability and the gut–liver axis, promoting chronic inflammation and the development of an immunosuppressive microenvironment conducive to tumorigenesis. Dysbiosis sustains a shift in gut-derived metabolites: increased levels of secondary BAs which downregulate FXR expression and induce aberrant cell proliferation; production of SCFAs determining genetic and epigenetic modifications, inducing changes in immune response and promoting cell proliferation; and oxidative stress leading to DNA damage and tissue remodeling.

## 3. Gut–Biliary–Liver Axis-Related Applications in Managing HBLC

### 3.1. Current Treatment Strategies: An Overview of Therapeutic Chances for Advanced HBLC Stages

#### 3.1.1. Hepatocellular Carcinoma

Nowadays, HCC represents a leading cause of death worldwide, severely affecting the prognosis and quality of life (QoL) in patients with advanced chronic liver disease (ACLD) [6]. Considering the potential onset of HCC even in less advanced stages of chronic liver disorders (CLDs) (viral and/or metabolic) or, as widely reported, even in the context of “healthy livers” via complex and incompletely clarified etiopathogenetic mechanisms, the clinical-epidemiological scenario appears broader and takes on further relevance [6].

Recently, scientific research efforts have allowed for the identification of specific molecular targets and the development of innovative systemic therapeutic regimens destined for patients with advanced HCC stages (Barcelona Clinic Liver Cancer—BCLC stage C) to improve survival and quality of life with a reasonable number of side effects [12].

Currently, the available therapeutic armamentarium includes tyrosine kinase inhibitors (TKIs) (Sorafenib and Lenvatinib) as well as several monoclonal antibodies (Mabs). Mabs, by targeting crucial molecules involved in the modulation of the immune response [programmed cell death ligand (PDL-1) (Atezolizumab)] and in the regulation of neo-angiogenesis [vascular endothelial growth factor-receptor (VEGF-R) (Bevacizumab)] in the tumor microenvironment, have dramatically revolutionized the therapeutic panorama [99]. In particular, the Atezolizumab-Bevacizumab therapeutic scheme is currently indicated as the first-line treatment for the majority of patients with advanced HCC (BCLC-C) [Child-Pugh class A and preserved performance status (Eastern Cooperative Oncology Group—ECOG PS 0-1)] [12]. Contraindications to Atezolizumab and/or Bevacizumab [99] allow for prescribing TKI-based regimens as a first-line treatment [12]. However, despite the encouraging results emerging from clinical studies, in real life, the response to these regimens is not homogeneous, with a non-negligible rate of patients failing multiple therapeutic lines [100].

#### 3.1.2. Cholangiocarcinoma

Cholangiocarcinoma embraces a large spectrum of different anatomically identified nosological entities, including ICC and ECC, which enclose perihilar CC (pCC) and distal CC (dCC) [101].

Traditional established standards of care represented by first-line (gemcitabine and cisplatin Gem/Cis ± Nab-Paclitaxel), second-line (FOLFOX regimen), and adjuvant (capecitabine) cytotoxic systemic chemotherapy (CSS) have been developed and are currently available for ICC, pCC, and dCC [101]. Unfortunately, traditional CSS regimens have been shown to minimally impact long-term outcomes in terms of overall survival amelioration, also appearing significantly burdened by CSS-related adverse events negatively influencing the QoL of CC-affected patients [101].

Therefore, in recent decades, the identification of pathogenetic targets and the development of “selective” therapeutic molecules have represented a relevant research challenge in this setting.

Regarding this, growing evidence has progressively highlighted non-genetic and genetic targets specifically featuring the advanced iCC subtype [101]. On the one hand, robust findings have highlighted a significative desmoplastic reaction with a rich tumor stroma that is actively involved in the iCC microenvironment, thus promoting interest in the immunotherapy research field [101].

Recent studies have revealed the pivotal role of the immune checkpoint blockade in advanced ICC, highlighting the potential benefits of adding Durvalumab (a Mab blocking the interaction of PD-L1 with the PD-1-Immune Check-point Inhibitor—ICI) to the traditional Gem/Cis scheme [102]. On the other, interestingly, iCC has been demonstrated as a potential “genetic cancer”, very frequently showing fibroblast growth factor receptor (FGFR) 2 fusions, BRAF gene aberrations, and isocitrate dehydrogenase 1 or 2 (IDH1 or 2) gain of function genetic mutations [103,104].

Relevantly, the identification of genetic alterations of FGFR2 or IDH1/2 has crucial implications in terms of treatment chances. Advanced iCC patients, after the evidence of progression on CSS and an adequate gene-typing revealing FGFR2 fusions/rearrangements or IDH1 mutations, may currently be included in dedicated clinical trials experimenting with targeted therapy [FGFR inhibitors or IDH inhibitors] and showing encouraging preliminary results [101,105,106].

**Figure 3:**
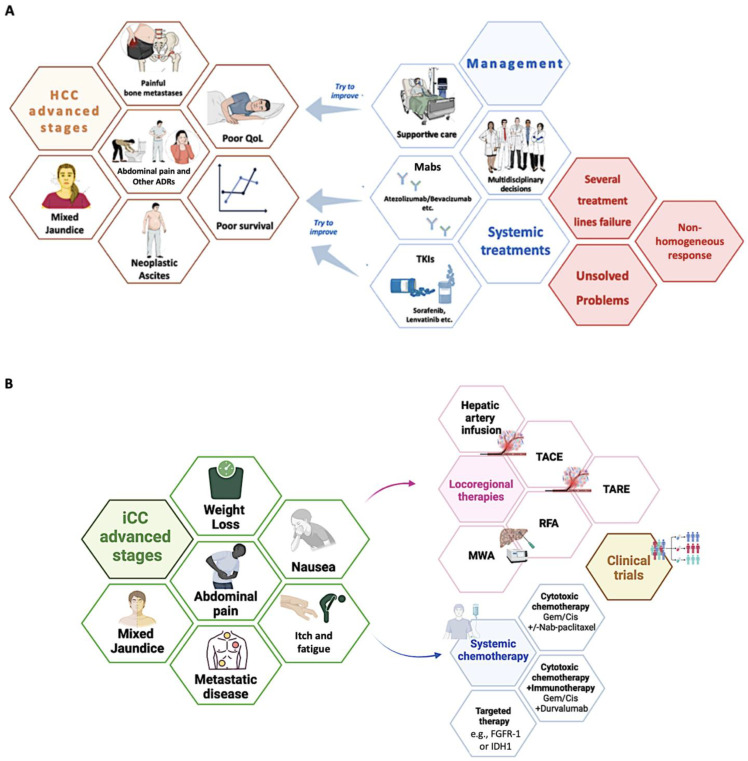
Proposed treatment strategies for the management of advanced hepatobiliary liver cancers based on patient- and disease-specific characteristics and relative management issues. (**A**)—Hepatocellular Carcinoma. Advanced-stage HCC-related manifestations and ADRs related to prescribed systemic therapies severely burden survival and QoL. The available therapeutic strategies for the management of HCC BCLC stage C include TKIs (Sorafenib and Lenvatinib; Mabs, which target crucial molecules involved in the modulation of the immune response (PDL-1: Atezolizumab and VEGF-R: Bevacizumab) in the tumor microenvironment; and supportive care. Abbreviations: HCC: hepatocellular carcinoma; QoL: quality of life; ADRs: adverse drug reactions; Mabs: monoclonal antibodies; TKIs: tyrosine kinase inhibitors; PDL-1: programmed cell death ligand-1; VEGF-R: vascular endothelial growth factor receptor. (**B**)—Cholangiocarcinoma. Advanced-stage iCC-related manifestations severely impact survival and QoL. The available therapeutic strategies for the management of advanced iCC include locoregional therapies, reserved for locally unresectable tumors involving both portal veins and/or the hepatic veins, an inadequate future liver remnant, and/or multiple tumors still confined to the liver, based on hepatic artery infusion, TACE, TARE, RFA, or MWA. Systemic chemotherapy, represented by cytotoxic chemotherapy (Gem/Cis +/−Nab-paclitaxel), cytotoxic chemotherapy + immunotherapy (Gem/Cis + Durvalumab), or targeted therapy (e.g., FGFR-I or IDH-I), is reserved for patients with liver and distant metastatic disease. Clinical trials are ongoing to evaluate the efficacy and safety of novel systemic strategies. Abbreviations: iCC: intrahepatic cholangiocarcinoma; TACE: trans-arterial chemoembolization; TARE: trans-arterial radioembolization; RFA: radiofrequency ablation; MWA: microwave ablation; Gem: gemcitabine; Cis: cisplatin; FGFR-I: fibroblast growth factor receptor inhibitors; IDH-I: isocitrate dehydrogenase inhibitors.

Figure 3 summarizes the therapeutic strategies and relative management issues for advanced HBLC (Figure 3A,B).

### 3.2. Microbiome–Gut–Biliary–Liver Axis-Related Potential Clinical Applications

#### 3.2.1. Gut Microbiota in Optimizing Early Diagnostic Processes in HBLC

In recent decades, among several strategies aimed at improving the prognosis of HBLC patients, major efforts have been focused on early identification of the disease for recognized high-risk individuals, thus ensuring a larger range of “curative”/“resolutive” therapeutic opportunities [11,101].

Concerning this, emerging research exploring the association of gut microbiota with HBLC has preliminarily suggested identifying microbiome biomarkers based on gut microbial alterations in CLD as a potentially useful translational approach to diagnose HCC at an early stage [94].

Regarding HCC, Ren et al. recently revealed a different fecal microbial diversity in patients with cirrhosis and early HCC compared with cirrhotic individuals without liver cancer, particularly evidencing an increased representation of the phylum *Actinobacteria* and 13 genera (such as *Parabacteroides* and *Gemmiger*), simultaneously with a decrease in butyrate-producing genera in early hepatic cancer compared with subjects presenting exclusively with liver cirrhosis [94].

In addition, in the same study, the optimal 30 microbial markers distinguishing non-HCC and early HCC cases were identified and subsequently successfully externally validated [94].

Altogether, this pioneering research first characterized the gut microbiome in HCC cases, established the diagnosis model, and validated the use of microbial markers, proposing gut microbiota-targeted biomarkers as a “personalized signature” and the candidate noninvasive approaches to diagnose HCC in the early stage.

As previously described, modern evidence suggesting the possible migration of oral microorganisms to the gut further complicates the above-presented pathogenetic scenario and thus the development of novel diagnostic strategies [50]. Concerning this, despite growing findings for potentially useful microbiome-related salivary markers in other disorders, after the encouraging results proposed by Lu et al. evidencing substantial microbiome diversity in the tongue coat of HCC patients [34], no research has specifically subsequently investigated this topic in the HCC setting, and no practical clinical applications have been derived.

Interestingly, very recently, Artificial Intelligence (AI)-based models have provided new evidence to this research field [107,108,109,110]. In particular, a recent study by Yang et al. aimed to identify features of both oral and intestinal microbiomes that could lead to the early detection of HCC through machine learning (ML) technology. They first distinguished different microbiome distributions in the microenvironment of the oral cave, tumoral tissue, and intestine. Significative differences in oral and gut microbiome composition were observed between HCC patients and healthy individuals, with a prominence of *Streptococcus* and *Shigella* spp. and *Escherichia coli*. The retrospective cohort was validated using ML and a random forest analysis in a prospective cohort, identifying ten oral and nine fecal bacterial genera capable of distinguishing HCC from healthy controls.

Remarkably, combining these features with serum alpha-fetoprotein (AFP) levels improved the model performance, providing an interesting new approach to be explored and applied in this line of research [111].

Regarding CC, several studies aimed to compare differences in gut microbiota between patients with CC and healthy patients to find potential non-invasive biomarkers for early diagnostics of CC [112,113,114,115]. Initially, the pattern of B-F-R genera (*Burkholderia-Caballeronia-Paraburkholderia*, *Faecalibacterium*, and *Ruminococcus-1*) was associated with stool samples as a diagnostic feature, even though the limitations of a single-center study with a restricted sample size significantly impacted the generalizable relevance of this pioneering research [113].

Another “microbiological signature” was evidenced by Deng et al. after a comprehensive analysis based on fecal 16S rRNA sequencing and clinical data in a cohort consisting of 40 healthy patients, 143 HCC patients, and 46 CC patients. In this research, a model based on eight bacterial genera (*Faecalibacterium*, *Klebsiella*, *Ruminococcus Gnavus* group, *Lactobacillus*, *Dorea*, *Veillonella*, *Burkholderia-Caballeronia-Paraburkholderia*, *Citrobacter*) appeared capable of discriminating patients with CC or HCC from healthy controls [115].

More recently, Zhang et al., aiming to systematically investigate the characteristics of the gut and bile microbiota in CC patients, enrolled 42 CC patients and 16 healthy controls, extracted DNA from fecal and bile samples, and performed 16S rRNA gene analysis [112].

The authors reported substantial differences in the species diversity and composition of microbial communities between CC individuals and healthy controls as well as a reduction in the relative representation of the phyla *Firmicutes* and *Actinobacteriota* simultaneously with increasing levels of *Proteobacteria* and *Bacteroidota* in patients with cancer. Moreover, in this research, the relative abundance of Klebsiella in the CC group was significantly higher than that in the controls, in contrast with the reduction in the relative abundance of Bifidobacterium. Finally, the Bifidobacterium/Klebsiella (B/K) ratio was found to be significantly decreased in the CC group compared with healthy individuals and was proposed as a novel diagnostic biomarker [112].

Comprehensively, these findings provide promising evidence supporting the potential use of gut microbiota-related markers as noninvasive tools for improving the diagnostic process of HBLC. Certainly, externally validating any novel proposed model on a real-world population appears to be crucially propaedeutic to the clinical application and implementation of this strategy. Integrating this into current clinical trials to develop combined algorithms able to identify early HBLC and predicting therapeutic responses and recurrence of this cancer constitute a paramount research challenge.

Table 2 summarizes the most relevant findings concerning specific microbiota composition patterns configuring a “tailored signature” potentially useful in the early diagnosis of HBLC (both for HCC and CC) in different settings (Table 2).

#### 3.2.2. Gut Microbiota as a Novel Tool in Predicting Treatment Response in Advanced HBLC

For neoplasms such as chemorefractory colorectal cancer (CRC) and non-chemoresponsive non-small cell lung cancer (NSCLC), a recent study suggested the association of the presence at baseline of the two butyrate-secreting microbial species (*Agathobacter M104/1* and *Blautia SR1/5*) with an improvement in progression-free survival (PFS) in patients treated with Mabs (avelumab and cetuximab) [116]. In contrast, concerning HBLC, there is severely limited evidence in the literature evaluating the influence of the pre-treatment intestinal microbial composition on the response to systemic therapeutic regimens, resulting in an incompletely defined scenario [117].

Concerning HCC, despite the encouraging findings elucidating the above-presented pathogenetic features, the investigation of individual microbiota as a signature predicting therapeutic response to systemic regimens continues to represent only a partially explored field [117].

Analysis of fecal samples from patients with unresectable HCC showed a significantly different microbiota composition between patients with radiology-proven objective responses (with a predominance of *Lachnoclostridium*, *Lachnospiraceae*, and *Veillonella*) and individuals with progressive disease (presenting an enrichment of *Prevotella 9*) [118]. Furthermore, the combination of increasing *Lachnoclostridium* and reducing *Prevotella 9* predicted superior OS in this setting [118].

In this context, a promising interesting target of investigation is represented by *Akkermansia muciniphila*, whose reduction seems to lead to an abundance of MDSCs with consequent suppression of the anti-tumoral immune response. Conversely, its supplementation has been reported to ameliorate liver injury, inflammation, and fibrosis and induce a significant “positive” shift in microbiota composition in murine models [35]. In particular, this reintroduction leads to an abundance of *Lachnospiraceae* and *Blautia*, which in turn have demonstrated anti-inflammatory properties due to the adequate production of SCFAs [38]. Scientific interest in *Akkermansia muciniphila* also derives from its possible role in favoring the immunotherapy response in mouse models. This mechanism involves increasing the recruitment of CCR9_+_ CXCR3_+_ CD4_+_ T-lymphocytes in cancer tissue depending on the stimulation of IL-12 from the dendritic cells [119].

In parallel with the efforts characterizing the gut microbiome signature and its relative adoption as a predictive treatment-response tool, another emerging innovative strategy to establish a clinical prognosis and predict the response to systemic therapies in HCC is represented by the genetic evaluation of factors involved in the regulation of gut-derived metabolic products. For this purpose, a Chinese study aimed to clarify the role of specific genes involved in the modulation of butyrate metabolism (BM). According to their results, these genes have proven to be promising biomarkers for use in this specific clinical setting to permit early identification of high-risk HCC patients and choose the best “tailored” therapeutic option [120].

Regarding CC, only two studies initially reported the results of patients with advanced-stage disease after failure of first-line treatment; in a total of 44 patients, the role of the gut microbiota in response to PD-1 antagonists was evaluated [121,122].

More recently, a pilot study revealed that abundance of the family *Ruminococcaceae* was inversely associated with chemotherapy response in ICC [123].

Interestingly, in addition to the chemotherapy setting, the microbiota composition and functioning has also been evaluated to provide potential advantages in predicting the response to surgical treatments, even in CC, where the evidence to date is decidedly scarcer. In this regard, to determine the impact on postoperative abdominal infections, *Bednarsch* et al. investigated bacterial colonization of the bile duct in the setting of pCC. *Enterococcus faecalis*, *Enterococcus faecium*, *Enterobacter cloacae*, and *Escherichia coli* were the most common bacterial species as well as the most common cause of postoperative abdominal infections [124], suggesting the potential improvement of surgical outcomes with future adaptation of an antibiotic prophylaxis.

Table 3 summarizes the most relevant evidence concerning specific microbiota composition patterns configuring a “tailored signature” that may be potentially useful in predicting the treatment response of HBLC (both for HCC and CC) (Table 3).

#### 3.2.3. Future Perspectives: Modulating Gut Microbiota as a Novel Therapeutic Strategy

As previously described, the microbiota plays pivotal roles in both health and disease conditions, impacting essential physiologic and pathologic processes, including metabolism, inflammation, and immunity response, appearing to be significantly involved in carcinogenesis and anti-cancer immune responses, with a particular reference to human HBLC [85].

This evidence has represented the rationale to guide modern research in exploring the modulation of the gut–liver axis and gut microbiome as a promising therapeutic frontier in the management of HBLC [33,125]. Concerning this, in recent decades, the progressive characterization of gut microbiota-related mechanisms involved in the pathogenesis of liver cancer has simultaneously sparked interest in using probiotics, antibiotics, prebiotics, symbiotics, dietary changes, and fecal transplants as therapeutic weapons to modulate intestinal flora composition and functioning and thus improve clinical outcomes [33,125].

Probiotics are live microorganisms that provide health benefits when consumed in adequate amounts, whose relevance in the context of HBLCs is due to certain species’ capability to adequately produce SCFAs (particularly butyrate and acetate), thus maintaining gut health, reducing inflammation, and supporting the immune system [126]. In line with this, the administration of specific SCFA-producing probiotics in modulating gut microbiota functioning has shown potential for improving the prognosis of HBLC-affected patients [67,93,126].

In mouse models, acetate significantly reduced the number and size of tumors, improving gut microbiome composition and intestinal barrier function, and acetate produced by *Bifidobacterium pseudolongum* has been shown to suppress NAFLD-associated HCC, suggesting the administration of probiotics adequately containing this species as a promising frontier in this setting [127].

Similar benefits have been described for the integration of butyrate-producing bacteria in patients with advanced HCC receiving an atezolizumab-bevacizumab regimen, reporting higher response and disease control rates in individuals consuming butyrate-producing bacteria during immunotherapy administration [128].

Relevantly, besides the immunotherapy setting, the gut microbiota has also been reported to modulate radiotherapy-associated antitumor immune responses against HCC via regulating the stimulator of interferon genes (STING) signaling, proposing the modulation of the microbiome as a potential enhancer of radiotherapy efficacy [129].

Furthermore, introducing bacteria from a donor to a host can represent a valid strategy to externally modulate the microbiota [129]. Currently, this is mainly achieved through FMT, where a physiological microbiota from carefully selected, healthy individuals is transferred [130].

A specific type of FMT is microbial ecosystem therapeutics (MET), which uses a defined mixture of pure living cultures of intestinal bacteria from a healthy donor’s stool sample. The transfer of microbiota can be done via endoscopy, enema, or oral capsules, though the optimal frequency, dose, and duration of FMT are still under debate [130].

So far, the effectiveness of FMT has been revealed in eradicating *Clostridium difficile* infections [131], whereas, in the field of oncology, initial investigations have focused on the anti-inflammatory effects of the transplant to prevent CLD and its progression to HCC [132].

In the setting of HBLC, transplanting the *Ruminococcaceae* family has been found to boost antitumor immunity by increasing the infiltration of tumors by CD8+ T cells [133]. These preliminary findings have opened doors to further investigations on FMT in modulating immune checkpoints, with various studies subsequently suggesting the role of this as a potentially valid strategy in enhancing response to immunotherapy in patients who are already responding to such treatments [117,133].

Moreover, cumulating research on patients with various types of tumors has indicated that several drugs (including, among others, antibiotics and proton pump inhibitors) can significantly affect clinical outcomes by altering the microbiota composition, suggesting medications as relevant modifiers of intestinal flora that should be taken into consideration in the optic of tailored treatment strategies [134,135]. In this context, the administration of metformin, an insulin-sensitizing drug known to impact the gut microbiota and contribute to increased levels of *Bifidobacterium* and *Akkermansia*, has been recently associated with a decreased incidence of HCC via regulating the FXR signaling pathway, ultimately sustaining anti-inflammatory effects [136]. Comparable interesting findings have been observed with the administration of aspirin and statins [117,137,138,139].

Altogether, these preliminary results appear to be even more relevant considering the widely used administration of these drugs in the setting of NAFLD, recently renamed MASLD [39], to manage cardiovascular (CV)-related comorbidities and increased CV risk observed in these patients [140].

In the aforementioned dysmetabolic setting, interesting results on the modulation of the gut–liver axis having a potentially functional translational role in the management of HBLC have been reported even for aliments, suggesting their implications in both preventive and therapeutic anticancer strategies via ameliorating underlying hepatic steatosis through relevant modifications in gut microbiota composition and functioning [141,142].

Regardless, besides the implications of these above-mentioned exogenous factors, a complete picture describing this context must take into consideration the role of even endogenous elements, with particular reference to the individual genetic background severely impacting pathogenesis and, consequently, management strategies of CLD-affected subjects [143,144].

## 4. Conclusions

In the era of Precision Medicine, where the creation of personalized therapeutic regimens and the identification of potential predictive markers of treatment response are priorities, the study of the possible influence of the intestinal microbiota and relative metabolites on the response to systemic treatment in patients with advanced HBLC represents an encouraging field of research to explore.

The future perspectives scenario on the modulation of the gut–liver axis in the development of novel therapies for HBLC patients emerges as a bifaces Janus: on the one hand, the simultaneous implications of several exogenous and endogenous factors offer a large spectrum of potentially useful translational targets, and on the other, this imbricated network contributes to the complexity of a pathogenetic network, remarking on the cruciality of further exploring the unclarified mechanisms. Certainly, integrating all these emerging approaches into the design of future clinical trials would represent a massive effort to significantly transition from bench to bedside in real life.

## Figures and Tables

**Table 1:** *Lactobacillus* spp. levels in different HBLC types and main relative functional implications.

Setting	*Lactobacillus* spp. Levels	Main Functional Repercussions	References
HCC patients	Reduced (↓↓↓) representation	Promoting inflammation via decreased SCFA production (mouse model) and inducing activation of hepatic TLR4/CXCL9 pathway	[66,67] 3/21/2025 10:11:00 AM.
Healthy subjects	Normal (↑) representation	Preserving intestinal integrity and contrasting inflammatory processes via SCFA production	[49,68,69]
CC patients	Enhanced (↑↑↑) representation	Impairing specific secondary BA production (correlation between bacterial representation and serum TUDCA levels)	[54]

HBLC: hepatobiliary liver cancer; HCC: hepatocellular carcinoma; CC: cholangiocarcinoma; SCFAs: short-chain fatty acids; TLR4: toll-like receptor; CXCL9: C-X-C motif chemokine ligand 9; BAs: bile acids; TUDCA: tauroursodeoxycholic acid. (↓↓↓): significantly reduced relative abundance; (↑↑↑): significantly increased relative abundance, (↑): increased relative abundance.

**Table 2:** Specific microbiota patterns and potential clinical applications in the diagnosis of HBLC.

Setting and Research Aims	Specific Microbiota Patterns (“Signature”)	Potential Clinical Applications	References
Discriminating patients with liver cirrhosis and early HCC from individuals exclusively presenting with liver cirrhosis	Gut microbiota: enhanced representation of *Actinobacteria*; increased levels of specific genera *Gemmiger*, *Parabacteroides*, *Paraprevotella*, *Clostridium_XVIII*, *Erysipelotrichaceae_incertae_sedis*, *Clostridium_XIVb*, *Collinsella*, *Butyricicoccus*, *Odoribacter Dorea*, *Acidaminococcus*, *Holdemania*, *Eggerthella*; decreased butyrate-producing species	Early identification of HCC in patients with liver cirrhosis	[94] 3/21/2025 10:11:00 AM.
Discriminating patients with HCC from healthy ones through a ML model	Gut and oral microbiota: enhanced representation of *Streptococcus*, *Shigella*, and *E*. *coli*	Early HCC detection; improvement of ML model performance after combining AFP serum levels	[111]
Discriminating patients with CC from healthy ones	Gut microbiota: enhanced representation of the specific genera *Burkholderia-Caballeronia-Paraburkholderia*, *Faecalibacterium*, and *Ruminococcus-1*, configuring the “B-F-R pattern”	Early CC detection	[113]
Discriminating patients with CC from healthy ones	Gut microbiota: reduced representation of phyla *Firmicutes* and *Actinobacteriota* simultaneously with increasing levels of *Proteobacteria* and *Bacteroidota*; relative abundance of *Klebsiella* in contrast with the reduction in *Bifidobacterium* [decreased *Bifidobacterium/Klebsiella* (B/K) ratio]	Early CC detection	[112]
Discriminating patients with HBLC (both HCC and CC) from healthy ones	Gut microbiota: enhanced representation of the specific genera *Faecalibacterium*, *Klebsiella*, *Ruminococcus Gnavus* group, *Lactobacillus*, *Dorea*, *Veillonella*, *Burkholderia-Caballeronia-Paraburkholderia*, *Citrobacter*	Early HBLC diagnosis	[115]

HBLC: hepatobiliary liver cancer; HCC: hepatocellular carcinoma; ML: machine learning; AFP: alpha-fetoprotein; CC: cholangiocarcinoma.

**Table 3:** Microbiota patterns and applications in predicting therapeutic response in advanced HBLC.

Setting and Research Aims	Specific Microbiota Patterns (“Signature”)	Potential Clinical Applications	References
Discriminating advanced HCC patients showing response (i.e., radiology-proven disease regression) from non-responders to systemic therapy	Gut microbiota: in responder patients, predominance of *Lachnoclostridium*, *Lachnospiraceae*, and *Veillonella*; in individuals with progressive disease, increased levels of *Prevotella 9* [increased *Lachnoclostridium/Prevotella 9* (L/P) ratio in long-term OS patients]	Predicting response to systemic therapy (disease progression and OS) in advanced HCC	[118] 3/21/2025 10:11:00 AM.
Identifying advanced HCC patients responding to immunotherapy	Gut microbiota: enhanced representation of *Akkermansia muciniphila* (in turn, promoting the increased relative abundance of SCFA-producer species *Lachnospiraceae* and *Blautia*)	Predicting immunotherapy response (disease progression) in advanced HCC	[35,38,119]
Identifying advanced ICC patients responding to immunotherapy	Gut microbiota: increased relative abundance of *Ruminococcaceae*	Predicting response to systemic therapy (disease progression) in advanced ICC	[123]
Estimating the risk of postoperative abdominal infections in advanced pCC	Bile duct microbiota: increased levels of *Enterococcus faecalis*, *Enterococcus faecium*, *Enterobacter cloacae*, and *Escherichia coli*	Predicting the outcomes in the post-surgical period in advanced CC	[124]

HBLC: hepatobiliary liver cancer; HCC: hepatocellular carcinoma; OS: overall survival; SCFAs: short-chain fatty acids; CC: cholangiocarcinoma; pCC: perihilar cholangiocarcinoma; ICC: intrahepatic cholangiocarcinoma. Conclusively, comprehensively investigating gut microbiota composition and relative microbial products intending to elaborate specifically tailored models predicting the response to therapeutic strategies in advanced HBLC represents a crucial research challenge with several socioeconomic health repercussions. In this sense, many steps forward have been made in this direction and, even if the feeling is that we have only seen the tip of the iceberg, it is essential to continue the efforts in this field of research to have valid tools capable of better guiding therapeutic choices and to define individual risks more precisely.

## Data Availability

No new data were created or analyzed in this study.

## Abbreviations

The following abbreviations are used in this manuscript:

ACLD	advanced chronic liver disease
AF	alpha-fetoprotein
ASV	amplicon sequence variants
BAs	bile acids
CC	cholangiocarcinoma
CLD	chronic liver disease
CMRFs	cardiometabolic risk factors
CRC	colorectal cancer
DAMPS	damage-associated molecular patterns
dCC	distal CC
EGF	epidermal growth factor
FGFR	fibroblast growth factor receptor
FMT	fecal microbiota transplant
FXR	farnesoid X receptor
GSH	glutathione
GVB	gut–vascular barrier
HBLC	hepatobiliary liver cancer
HCC	hepatocellular carcinoma
HDAC	disrupting histone deacetylase
HMGB1	high-mobility group box 1
HNE	4-hydroxy-trans-2-nonenal
ICC	intrahepatic cholangiocarcinoma
IDH1 or 2	isocitrate dehydrogenase 1 or 2
ILC 3	group 3 innate lymphoid cells
IR	insulin resistance
JAMs	junctional adhesion molecules
LC	liver cirrhosis
LPS	lipopolysaccharide
Mab	monoclonal antibodies
ML	machine learning
mMDSC	monocytic myeloid-derived suppressor cells
MASLD	Metabolic Dysfunction-Associated Steatotic Liver Disease
NAFLD	non-alcoholic fatty liver disease
NKT	natural killer T
NSCLC	non-small cell lung cancer
PAMPs	pathogen-associated molecular patterns
PC	pancreatic cancer
pCC	perihilar cholangiocarcinoma
PDL-1	programmed cell death ligand 1
PFS	progression-free survival
PKC	protein kinase C
PKA	protein kinase A
PMN-MDSCs	polymorphonuclear myeloid-derived suppressor cells
PSC	primary sclerosing cholangitis
QoL	quality of life
ROM	reactive oxygen metabolite
ROS	reactive oxygen species
rRNA	ribosomal RNA
SCFAs	short-chain fatty acids
SOD	superoxide dismutase
STING	stimulator of interferon genes
TKI	tyrosine kinase inhibitors
TJs	tight junctions
TLR	toll-like receptor
TUDCA	tauroursodeoxycholic acid
VEGF-R	vascular endothelial growth factor receptor
VI	vascular invasion
ZO	zonula occludens
